# Polysaccharide-Encapsulated Lauraceae Extract Complex Coating Conferring Antimicrobial Properties to Polypropylene Surfaces

**DOI:** 10.1021/acsomega.5c00665

**Published:** 2025-06-03

**Authors:** Tuyet-Nhi Do, Po-Hsin Lee, Tsung-Lin Tsai, Ping-Ching Wu

**Affiliations:** † Department of Biomedical Engineering, College of Engineering, 34912 National Cheng Kung University, Tainan 701, Taiwan; ‡ Meet Tec. Co., Ltd., Tainan 710, Taiwan; § Center of Applied Nanomedicine, 34912 National Cheng Kung University, Tainan 701, Taiwan; ∥ Department of Oncology, National Cheng Kung University Hospital, College of Medicine, 34912 National Cheng Kung University, Tainan 701, Taiwan; ⊥ Institute of Oral Medicine and Department of Stomatology, National Cheng Kung University Hospital, College of Medicine, 34912 National Cheng Kung University, Tainan 701, Taiwan; # Medical Device Innovation Center, Taiwan Innovation Center of Medical Devices and Technology, National Cheng Kung University Hospital, 34912 National Cheng Kung University, Tainan 701, Taiwan

## Abstract

The rise in antimicrobial resistance (AMR) pathogens necessitates innovative approaches for infection control, particularly in medical device applications. Moreover, simultaneously ensuring sustained antimicrobial activity in biomaterials and minimizing potential resistance development remain critical aspects that lack new strategies. This study presents the development and application of an innovative antimicrobial agent, that is, a multifunctional polysaccharide-encapsulated Lauraceae extract complex (PLEC) coating designed to enhance the antimicrobial efficacy and compatibility of polypropylene surfaces. The study findings indicate that the PLEC coating has potent antibacterial and antifungal characteristics, preventing a wide range of infections. A comprehensive analysis revealed significant reductions in bacterial colonies, including colonies of Gram-negative bacteria (Escherichia coli and Pseudomonas aeruginosa), Gram-positive bacteria (Staphylococcus aureus and Streptococcus mitis), and various fungal strains. Moreover, cytotoxicity evaluations confirm the nontoxic nature of the coatings, ensuring their safety for medical use. This study demonstrates that multifunctional antimicrobial PLEC coatings offer a significant advancement in infection control strategies, providing an effective solution to the growing problem of AMR. The developed approach can be beneficial to the healthcare industry and society by reducing infection risks and treatment costs, ultimately improving clinical effectiveness and enhancing the efficacy of infection control measures in medical applications.

## Graphical abstract

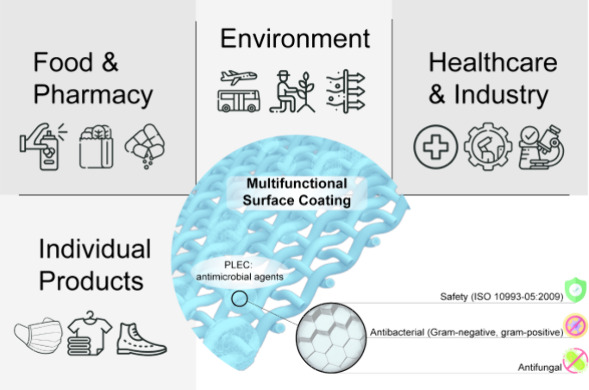

## Introduction

The increasing prevalence of antimicrobial resistance (AMR) bacteria is becoming a major global health concern.^1^ As the efficacy of traditional antimicrobial declines, infections caused by multidrug-resistant pathogens increase and spread rapidly, complicating treatment protocols and elevating public health risks.^2^ In healthcare settings, resistant infections increase morbidity, mortality, and healthcare costs.^1^ Therefore, novel antibacterial materials that provide effective and sustainable protection without increasing resistance are urgently needed for clinical application.^2,3^

However, the development of new antimicrobial agents is challenging. Many current solutions face difficulty balancing antimicrobial effectiveness, safety, durability, and cost-effectiveness. Additionally, long-term efficacy, which is crucial for preventing infections in medical and industrial environments, is often insufficient.^3^ Certain antibacterial materials promote the development of microbial resistance, which complicates infection control.^2^

Polypropylene (PP) substrates are widely used as biomaterials, owing to their biocompatibility, mechanical durability, and chemical resistance. However, their susceptibility to microbial contamination presents a considerable risk,^4,5^ where surface-related infections can have severe complications—particularly in clinical applications.^6,7^ To address these challenges, the present study presents the development of a multifunctional antimicrobial coating for PP substrates. The membrane effectively kills Gram-negative and Gram-positive bacteria and inhibits fungal growth, enabling safe and prolonged usage. Coating techniques provide a practical and scalable solution to improve the effectiveness of existing substrates rather than creating new antibacterial materials from scratch.^8,9^ Recent studies indicate that nanoparticles and polymer coatings could impart antimicrobial characteristics to PP.^6,7^ Research on multifunctional coatings that are biocompatible and simultaneously exhibit antimicrobial, antifungal, and filtration properties is in high demand. Formulations suitable for sensitive environments and healthcare applications require further research.

Various antimicrobial coating strategies have been explored to reduce the level of pathogen transmission on polymeric surfaces. Polyurethane coatings functionalized with quaternary ammonium groups (e.g., MPU3-D) offer improved biocompatibility and water dispersibility, yet their efficacy depends on high methylation levels and is limited under alkaline conditions.^10^ Metal-based systems, such as CuONPs/TiO_2_ nanocomposites, exhibit excellent bactericidal activity and superhydrophobicity.^11^ However, concerns over cytotoxicity, environmental accumulation, and resistance development remain unresolved.^11^ Quaternized chitosan derivatives provide a biodegradable alternative with increased water solubility and a positive charge density. Despite their potential, they often limit their antibacterial activity to Gram-positive strains, and their effectiveness hinges on their application and cross-linking methods.^12^ Many current systems rely on synthetic modifiers or metals, raising further safety and sustainability issues.^13^ In response, recent efforts have focused on reducing nanotoxicity and limiting metal nanoparticle release in biomedical coatings.^14,15^ At the same time, the global shift toward eco-conscious design and net-zero goals has driven interest in naturally derived, biodegradable materials sourced from renewable resources as safer and more sustainable alternatives.^16^

Our study introduces a polysaccharide-encapsulated Lauraceae extract complex (PLEC) as a natural, broad-spectrum antimicrobial coating. PLEC integrates bioactive plant-derived compounds within a biopolymer matrix, eliminating the need for metal ions or synthetic biocides. The coating demonstrates vigorous antibacterial activity against Gram-positive and Gram-negative bacteria, sustained antifungal efficacy, and confirmed cytocompatibility. The formulation is entirely water-based and applied to polypropylene substrates by using thermal temperature coating methods, offering scalability and environmental safety. The PLEC formulation overcomes key metal- and polymer-based systems’ limitations by addressing both efficacy and safety, positioning it as a multifunctional and sustainable alternative for antimicrobial applications in healthcare and consumer products.

We leveraged a thermal coating technique^17^ to produce a durable membrane with potent antibacterial and antifungal properties.^18^ We integrated an antimicrobial inclusion complex from the PLEC as an antimicrobial coating for PP substrates that ensures biocompatibility, addressing a key requirement for clinical biomaterials.^2,3,9^ The adaptability of the membrane enables its application in various fields, including healthcare, environmental protection, food safety, and pharmaceuticals—reducing infection risks, improving treatment outcomes, and providing cost-efficient solutions for industry.^2,19^

This study demonstrates the antimicrobial effectiveness of the PLEC coating, revealing its ability to disrupt and prevent the growth of various bacterial strains.^20–22^ Our findings hold significant implications for functional biomaterials and protective consumer applications, particularly the urgent need for biocompatible and durable antimicrobial materials.^21^

## Experimental Section

### Materials

The following materials were used: β-cyclodextrin (βCD, Sigma–Aldrich), cinnamon oil (Sail brand, 103007), poly­(vinyl alcohol) (PVA, Mw 31,000–50 000 Da, Sigma–Aldrich), polypropylene (PP) nonwoven fabric (Full Men Fabric Co., Ltd., Taiwan), American bacteriological agar (Condalab cat. 1802), Brain Heart Infusion Broth (BD 237500), Difco LB Broth (Miller 244620), Difco LB agar (Miller 244520), LB agar (Miller XR-IMK110283), tryptic soy broth (BD cat. 211825), tryptone (Condalab cat. 1612), yeast extract (Condalab cat. 1702), high-glucose Dulbecco’s modified eagle’s medium (HG-DMEM, Gibco), sodium bicarbonate (Sigma–Aldrich), Cell Counting Kit-8 (CCK-8, Dojindo), fetal bovine serum (FBS, Gibco), 0.5% trypsin-EDTA 10× solution (GeneDireX/Simply), antibiotic-antimycotic solution (GeneDireX), hydrochloric acid (HCl, Sigma–Aldrich), 0.4% trypan blue solution (Gibco, 15250061), 0.33% neutral red solution (Sigma–Aldrich, N2889), agarose (GeneDireX, MB755-0100), Tween 20 (Sigma, P1379), and a FilmTracer LIVE/DEAD Biofilm Viability Kit (Invitrogen, L10316).

### Methods

#### Preparation and Characteristics of the Membrane Coating

We prepared the PLEC coating solution by forming an inclusion complex between cinnamon oil and βCD (CβCD), following a modified encapsulation method described by Bhandari.^23^ Specifically, βCD (9 g) was mixed with deionized water (100 mL) and stirred with a magnet at 90 °C for 30 min until the complete dissolution of βCD. Cinnamon oil was slowly added to the hot βCD solution in a 1:3 (w/w) ratio while the mixture was constantly stirred for 1–1.5 h. The mixture was then stirred without heating, while the temperature decreased to 25 ± 1 °C with constant agitation. Subsequently, the solution was stored at 4 °C overnight, followed by vacuum filtration under cold conditions. The precipitate was collected, dried at 50 °C for 24 h in a hot air dryer system, and stored in airtight glass containers at 4 °C. The PVA solution was prepared by dissolving PVA in deionized water at a concentration of 10% (w/v), and stirred at 90 ± 1 °C until fully dissolved. The solution was then autoclaved at 121 °C for 20 min and cooled to room temperature before being diluted to a working concentration of 1% (w/v). The final PLEC coating solutions were created by adding 1, 2, and 3% (v/v) CβCD dissolved in a PVA solvent. We sonicated the PLEC coating solution to induce a spontaneous reaction within 10 min. We prepared the membrane coating via a heat-dip coating technique.^17^ Fine PP substrates were dip-coated in the prepared coating solutions with different concentrations at a ratio of 0.04 mL/cm^2^ and then placed in an oven at 60 °C for 20 ± 5 min until fully dried. We characterized the PLEC-coated PP membrane structures and fiber diameters, as well as those of an uncoated PP using high-resolution field-emission scanning electron microscopy (FE-SEM, SU8010, Hitachi). We evaluated the nonviable particle retention or filtration efficiency by using the particle filtration efficiency (PFE) test. Additionally, we assessed the electrostatic stability (ES) of the samples.^24,25^

#### Bactericidal Efficiency

The bactericidal efficiency of the PLEC-coated membrane was investigated via the American Association of Textile Chemists and Colorists (AATCC 100) method.^26,27^ The process involved culturing the PLEC-coated membrane with a standard inoculum at a bacterial concentration of 10^5^ CFU/mL (OD_600_ = 0.5–0.6) to test for Escherichia coli (E. coli, ATCC 33572). For Staphylococcus aureus (S. aureus, ATCC 21351), both clinical strains were generated using LB Broth, and Pseudomonas aeruginosa (P. aeruginosa, ATCC 27853) and Streptococcus mitis (S. mitis, ATCC 49456) were cultured by utilizing tryptic soy broth. The turbidities of the samples were determined at a wavelength of 600 nm by using an ultraviolet (UV)–visible spectrophotometer. The test microorganisms used in the study included E. coli and P. aeruginosa, as representative Gram-negative bacteria, and S. aureus and S. mitis, as representative Gram-positive bacteria. The AATCC 100 quantitative test method was used to evaluate the antibacterial effectiveness.^18,26^ One milliliter of bacterial inoculum was employed for each sample, which were subsequently cultured at 37 °C for 20–24 h under CO_2_ atmosphere. Following incubation, the final cell concentrations in the control and test samples were calculated by measuring the number of viable cells in CFU/mL. The agar plates were incubated at 37 °C for 20–24 h. Following the incubation period, the number of colonies present on each dilution plate was counted and correlative calculations were performed.^27–30^

The following formula was used to calculate the number of colony-forming units:

$$\mathrm{CFU}/\mathrm{mL}=\frac{\mathrm{number}\;\mathrm{of}\;\mathrm{colonies}\times \mathrm{dilution}\;\mathrm{factor}}{\mathrm{volume}\;\mathrm{of}\;\mathrm{culture}\;\mathrm{plate}}$$

#### Antifungal Susceptibility

In the initial evaluation to assess the antifungal properties of the antimicrobial blend compounds, a 2% PLEC coating solution (PLEC-sol 2%) was embedded in the surface of bread and compared with blank (1% PVA) and control groups.^31^ During the first 3 days, sterile water (20 mL) was added to each 49.5 ± 1.3 g sample. We homogenized the samples and maintained the same lighting, temperature, and moisture conditions.^32,33^ Assays were performed three times. To assess the total fungal growth area in the bread, we collected data on days 0, 3, 7, 14, and 21 of the experiment.^34^ The samples were routinely examined and photographed. ImageJ software 1.53a was used to process and analyze the images.^31,35,36^

#### Biocompatibility

Cytotoxicity tests were conducted following the ISO 10993-5:2009 guidelines.^37,38^

##### Cell Viability

A series of PLEC-sol 2% concentrations ranging from a dilution of 1 × 10^–5^ to a concentration of 1 × 10^0^ were assessed for toxicity via in vitro tests using the mouse fibroblast line NIH/3T3 (ATCC number: CRL-1658). Cells were cultured under physiological conditions in an incubator (37 °C, 5% CO_2_, and 95% humidity). NIH/3T3 cells were harvested and cultured in 96-well plates at a concentration of 5000 cells/100 μL in each well. Different concentrations of PLEC-sol 2% were added, including HG-DMEM with 10% FBS (HG-DMEM complete medium) as a control, with the same volume of 100 μL per well. The number of viable cells in a population was measured 24 ± 2 h after treatment. The number of viable cells was quantified via the CCK-8 assay.^18,39,40^ Before measurement, 10 μL of the CCK-8 kit mixture was added to each well for 2 h. The absorbance of the cells was measured at 450 nm using a microplate reader.^38,41^ Results were obtained by dividing the cell viability of the treatment group by that of the control group using various doses of PLEC-sol 2% at a series of dilutions. Each group (*n* = 3) was quantitatively tested.

##### Agar Diffusion Test

For the in vitro tests, HG-DMEM complete medium was used as the extractant. The ratio of the PLEC-coated 2% membrane to the extractant was 100 mg/mL, and the mixture was incubated at 37 ± 1 °C for 24 ± 2 h with constant agitation at 200 rpm by using a stir bar. Microscopic observation of the cell morphology and reactivity zones was performed by using a neutral red assay. The assay was performed using the disc diffusion technique of the qualitative agar diffusion test.^26,39^ The NIH/3T3 (ATCC number: CRL-1658) and Vero (ATCC CCL-81) (5 × 10^4^ cells/mL) cell lines were cultured at 2 mL/well in a six-well plate with complete HG-DMEM. The cells were then incubated at 37 ± 1 °C under humidified atmosphere containing 5 ± 0.5% CO_2_ for at least 24 h until the cultures had grown to approximately subconfluence. The culture medium in each well was then replaced with agar (2 mL). The cultures in the agar media were incubated at 25 ± 1 °C for the solidification of well-mixed agar media, which included equal volumes of the HG-DMEM complete medium and 1% agarose solution. Subsequently, the culture medium in each well was replaced with agar (2 mL) and the mixture incubated at 25 ± 1 °C for solidification. The 120 mm^2^ PP substrates were sterilized at 121 °C by autoclaving. They were then exposed to UV light overnight before being placed under the agar media surface for the direct-contact testing of each group, except the control group. HG-DMEM complete medium, 5% Tween 20, and PLEC-coated 2% were extracted onto sterile PP substrates on the surface of the agar media for the negative, positive control, and PLEC-coated 2% groups, respectively. The cultures were then incubated at 37 ± 1 °C under a humidified atmosphere containing 5% ± 0.5% CO_2_ for 24–26 h. Each group was quantitatively tested using 2 mL of a 0.005% neutral red solution. The cultures were further incubated at 37 ± 1 °C under a humidified atmosphere containing 5% ± 0.5% CO_2_ for 2.5 ± 0.5 h. The cells were examined under a microscope to evaluate the cytotoxic effects based on the degree of reactivity. The scores of each test were averaged to provide a final assessment of biological reactivity.^25,37,42^ The experiment was repeated three times to ensure reliability.

#### Bacterial Membrane Integrity

##### Transmission Electron Microscopy (TEM)

The morphologies of both the negative and positive strains were examined by using TEM (JEM-1400, JEOL, USA). Primary bacterial cultures were prepared in sterilized broth via incubation at 37 °C for 12 h. The samples were then incubated at 37 °C for 1 h with a 10^5^ CFU/mL bacterial culture. The PLEC-coated 2%, PLEC-sol 2%, blank, and control bacterial cells were centrifuged at 5000 rpm for 5 min at 4 °C to prepare the resin-embedded samples. The samples were completely dispersed and diluted with deionized water. They were fixed for 1 min at 25 ± 1 °C with a 2% uranyl acetate solution, placed on a copper grid, and subjected to negative staining. Subsequently, the samples were examined under 30,000 × magnification, and a selected section was enlarged.

##### Live/Dead Bacterial Staining

The standard concentrations of the cultured bacteria were determined to be OD_600_ = 0.8 and 1 for E. coli and S. aureus, respectively. For each group, the volume of bacteria used for testing was 1.0 ± 0.1 mL, and the samples were incubated for 30 min. The specified bacteria were rinsed twice with 1× phosphate-buffered saline before staining, following the SYTO 9 and propidium iodide (PI) staining protocols for the Film Tracer LIVE/DEAD Biofilm Viability Kit. Finally, the samples were imaged using confocal laser scanning microscopy (Nikon-ECLIPSE Ti2-E, Japan) to obtain fluorescence images.

#### Quantification and Statistics

The data were graphically visualized by using GraphPad Prism version 10.0.3 (217). All data were statistically analyzed using either Student’s *t* test or one-way analysis of variance (ANOVA) followed by Dunnett’s multiple comparison test or two-way ANOVA followed by Tukey’s multiple comparison test, with a single pooled variance to establish the significance between data points. All of the data are presented as the mean ± standard error of the mean (SEM).

## Results and Discussion

### Characteristics of Materials

The surface characteristics of the PLEC-coated and uncoated PP substrates were analyzed by using FE-SEM. The coating process significantly changed the surface morphology (Figure 1a). The fiber diameter increased from 5.11 ± 0.8 μm in the uncoated membrane to 6.92 ± 1.2 μm in the coated membrane (Figure 1b), confirming the presence of the PLEC coating. Subsequently, the PFE and ES of the filter were assessed to verify the effect of the coating. The PFE of the coated filter was evaluated at an airflow rate of 85 L/min. The coated membrane had a filtration efficiency higher than that of the uncoated filter (Figure 1c). Furthermore, the ES of the coated filter (Figure 1d) enabled the membrane to retain its filtration efficiency over time, even in dynamic environments. These properties are crucial for preventing bacterial and particulate contamination in medical applications—particularly in devices, such as surgical masks and filtration systems.

**Figure 1:**
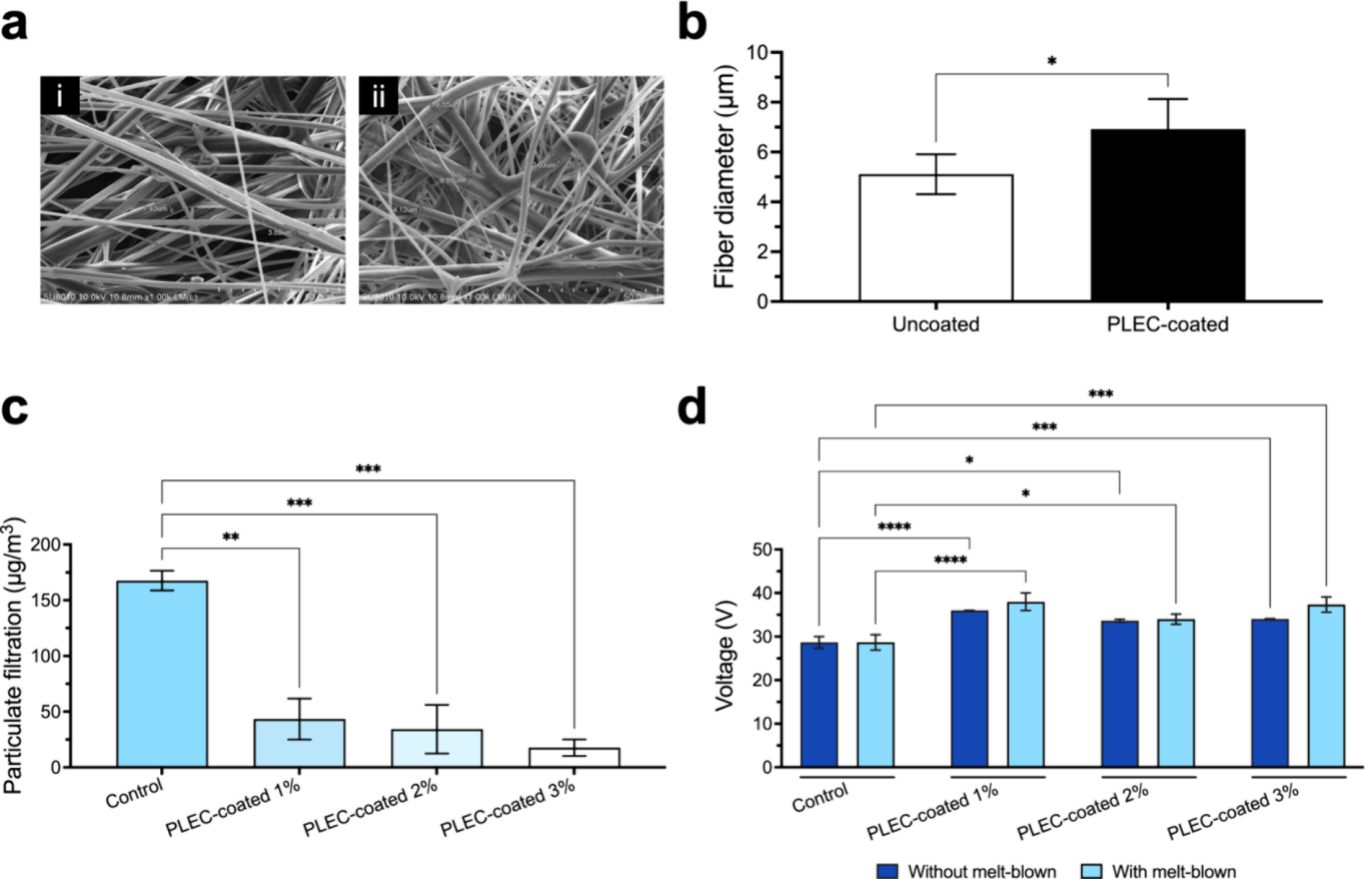
Characterization of the membrane coating. Structural and filtration properties of the uncoated PP substrate and PLEC-coated membrane were evaluated. (a) FE-SEM images revealed the surface morphology of the (i) uncoated and (ii) PLEC-coated membranes, and (b) the fiber diameter ranged from 5.11 ± 0.8 to 6.92 ± 1.2 μm. (c) PFE assessment using a closed box with suspended particles (1000 μg/m^3^) by the control and PLEC-coated membranes for 1 min. Significant differences compared with the control substrates were observed. (d) ES of the control and PLEC-coated membranes with and without a melt-blown layer compared, revealing that the coating process had a profound impact on the electrostatic properties compared to those of the control groups. *p < 0.05, **p < 0.01, ***p < 0.001, and ****p < 0.0001. Values expressed as mean ± SEM (*n* = 3).

PP is widely used in these applications for its favorable mechanical properties and biocompatibility. However, its lack of inherent antimicrobial properties makes it vulnerable to microbial contamination. Therefore, enhancing the surface functionality of PP via antimicrobial coatings has become a growing area of research. The PLEC coating developed in this study improves the antibacterial performance of PP and maintains its filtration efficiency, offering a promising solution for infection control in healthcare and protective equipment.^43,44^

### Antibacterial Properties

The antibacterial efficacy of the PLEC-coated membranes was rigorously tested against Gram-negative (E. coli and P. aeruginosa) and Gram-positive bacteria (S. aureus and S. mitis). Figure 2a shows the colony morphology and bacterial counts, initialized at 1 × 10^5^ CFU/mL, after 24 h of incubation. The PLEC-coated membranes exhibited significantly reduced numbers of bacterial colonies compared with the uncoated and control membranes. The antibacterial effect was found to be within the range from the lowest concentration (PLEC-coated 1%) to the highest concentration (PLEC-coated 3%), inhibiting the reproduction of both Gram-negative and Gram-positive bacteria (Figure 2b). Further evaluation using a modified AATCC protocol with a shortened 16 h incubation period and a higher initial inoculum of 10^7^ CFU/mL, as illustrated in Figure S1, confirmed the concentration-dependent bactericidal efficacy of PLEC. The PLEC-coated 2% group was identified as the minimum inhibitory concentration, effectively suppressing visible colony formation across all tested strains. This additional validation reinforces the reliability of the antibacterial effect and supports the consideration of PLEC-coated 2% as a suitable candidate for clinical application. The sustained release of PLEC from the membrane coating ensured the continuous inhibition of bacterial growth, which is essential for long-term infection prevention in medical devices. This prolonged release mechanism minimizes the need for the frequent reapplication of antimicrobial agents, rendering it a practical solution for clinical environments in which devices are used for extended periods.

**Figure 2:**
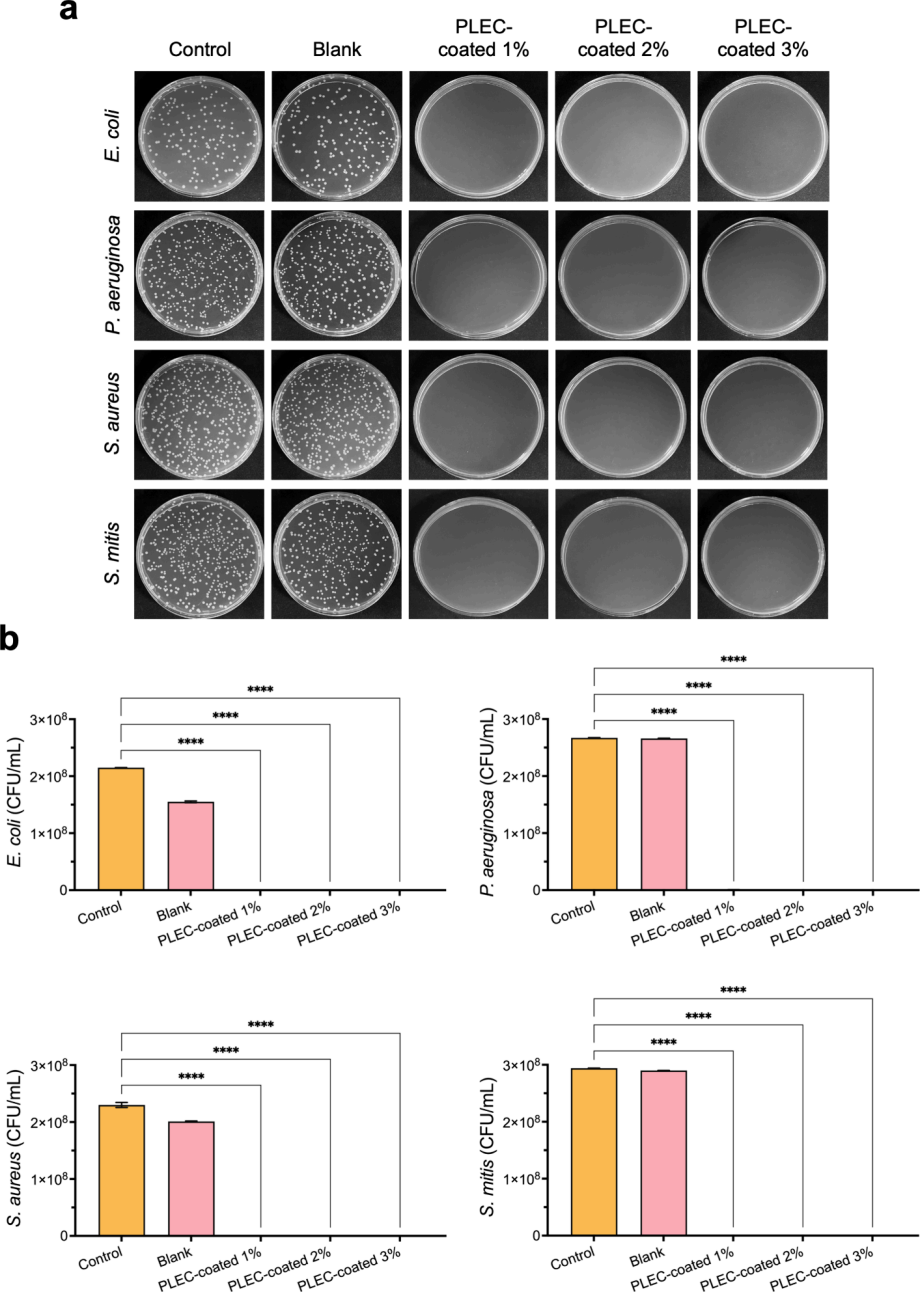
Inhibition of bacterial growth by a membrane coating. Antibacterial efficacy of the PLEC-coated membranes tested against both Gram-negative and Gram-positive bacteria. (a) Colony growth of Gram-negative (E. coli, P. aeruginosa) and Gram-positive (S. aureus, S. mitis) bacteria after 24 h of incubation with PLEC-coated membranes compared with control and blank groups. (b) A significant reduction in bacterial colonies evident even at the lowest concentration of the PLEC-coated 1%, indicating that the membrane coating effectively inhibited both Gram-negative and Gram-positive bacteria. **** *p* < 0.0001 compared with the control group. Values expressed as mean ± SEM (*n* = 4).

### Antifungal Susceptibility

In addition to the antibacterial activity, PLEC-sol was tested for antifungal efficacy. Fungal growth was monitored for 21 days using bread slices. Figure 3a shows top and bottom views of the fungal growth on the bread slices with the groups indicated. We quantified the fungal area using ImageJ until 21 d. PLEC-sol 2% significantly inhibited fungal proliferation, as evidenced by a reduction in the fungal area relative to that in the control and blank groups (Figure 3b). The fungal area on PLEC-sol 2% (85.99 ± 0.21, 92.32 ± 0.17, and 95.13 ± 0.51 mm^2^) slowly increased and was less than that on the control group (129.29 ± 0.20, 143.99 ± 1.39, and 148.46 ± 0.46 mm^2^) from 7 to 21 d, demonstrating the ability of the PLEC-sol 2% coating to inhibit fungal growth over an extended period (Figure 3c). The significant reduction in the fungal area suggests that the coatings can provide broad-spectrum antimicrobial protection—particularly in healthcare settings.^45,46^ Thus, the developed coatings offer a dual protective effect by preventing bacterial and fungal contamination.

**Figure 3:**
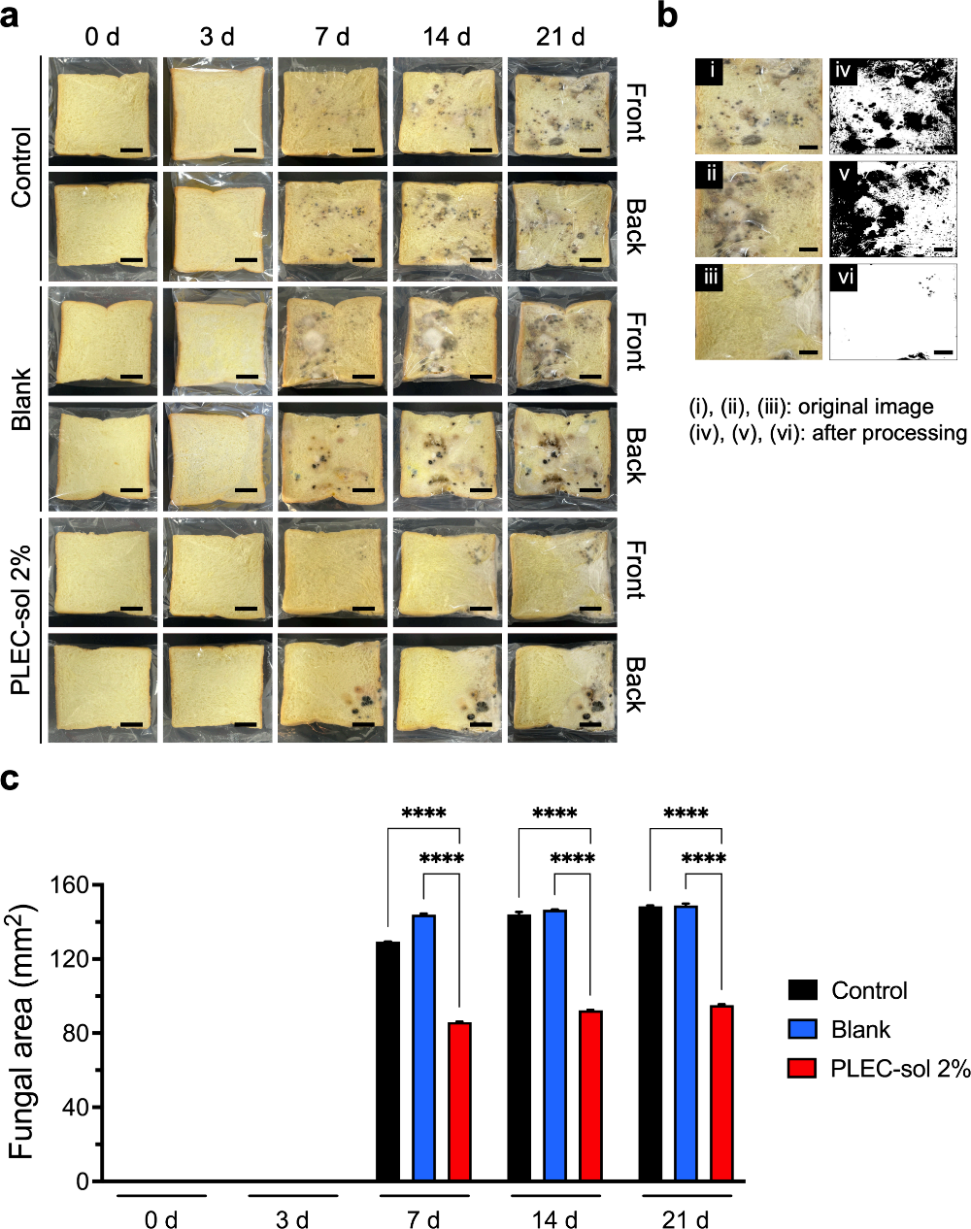
Antifungal susceptibility of PLEC-sol. Antifungal properties of the membrane coating were assessed by examining the fungal growth on the bread slices at 0, 3, 7, 14, and 21 d. (a) Visual observations of fungal growth on the bread slices embedded with PLEC-sol 2% on both sides at different points of time. (b) Quantitative analysis of the fungal area before and after processing using ImageJ, revealing a notable reduction in fungal proliferation in samples treated with PLEC-sol at 2%. (c) Fungal growth area measured at 0, 3, 7, 14, and 21 d, revealing significant inhibition of fungal growth by PLEC-sol. **** *p* < 0.0001 compared to the control and blank groups. Values expressed as mean ± SEM (*n* = 3).

### Biocompatibility

We performed a CCK-8 assay using NIH/3T3 mouse fibroblasts to evaluate the cytotoxicity of the PLEC-sol. The cell viability was assayed at different concentrations of PLEC-sol of 2%. The results of the CCK-8 assay indicated that the PLEC coatings had low cytotoxicity to NIH/3T3 cells, as evidenced by high cell viability at different concentrations of PLEC-sol 2% (Figure 4a). Furthermore, the IC_50_ value was calculated, which indicated that the coating was nontoxic to normal cells at the tested concentrations (Figure 4b). These results confirm that PLEC-sol is safe for medical applications. Maintaining high cell viability while providing antimicrobial protection is critical for materials used in medical devices because it ensures that the coatings do not harm the surrounding healthy tissue during use.

**Figure 4:**
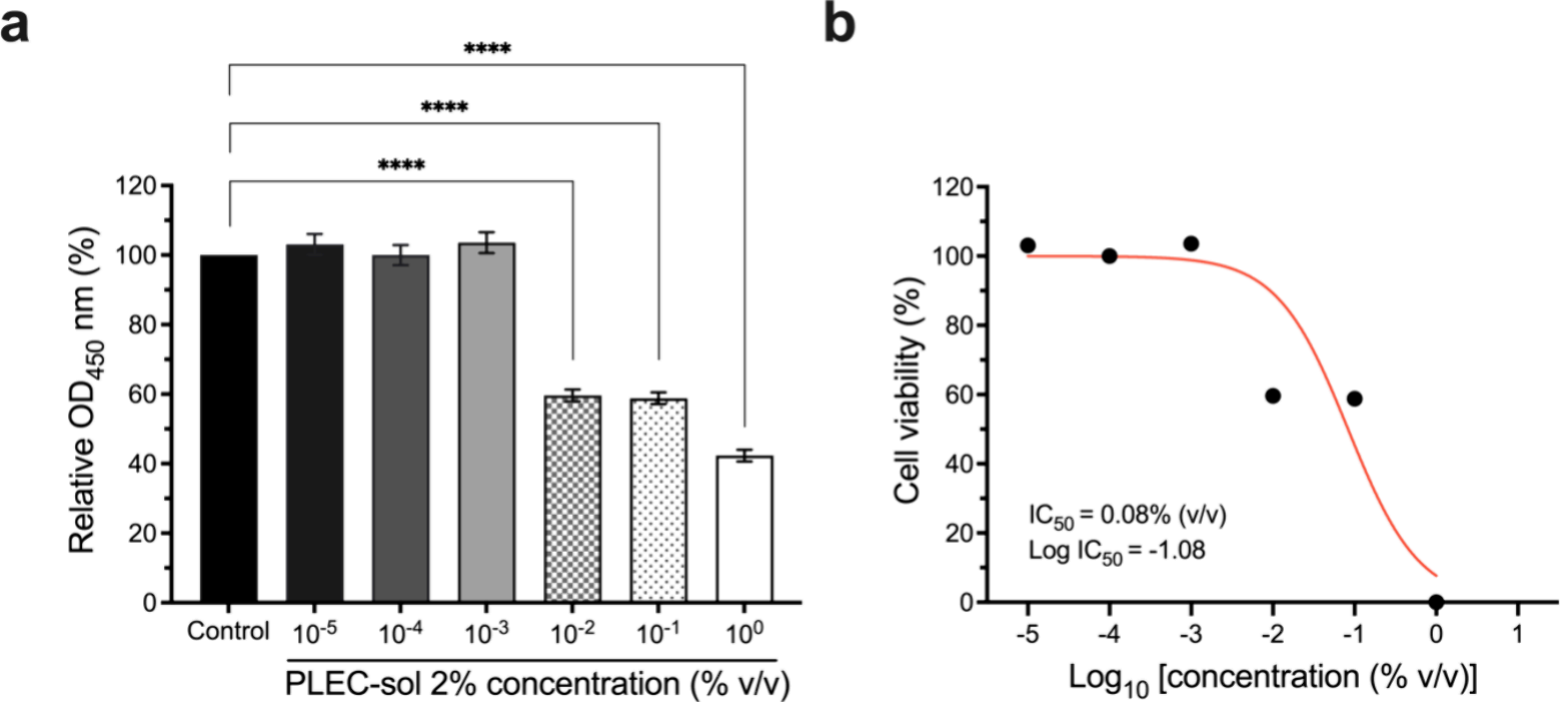
Cytotoxicity of PLEC-sol assessed using the CCK-8 assay in NIH/3T3 cells. (a) Cytotoxicity in NIH/3T3 mouse fibroblasts treated with various concentrations of PLEC-sol measured using the CCK-8 assay. Cell viability of 42.31% ± 1.67% up to 103.05% ± 2.97% at a series of diluted concentrations indicated minimal cytotoxicity. (b) IC_50_ value graph confirming that the coated membranes are safe for clinical use, with IC_50_ values exceeding 0.08% (v/v). ****p < 0.0001 compared with the control group. Values expressed as mean ± SEM (*n* = 3).

Further biocompatibility testing was conducted using an agar diffusion test with neutral red staining, which assessed the potential cytotoxicity in direct contact with the cells. The results indicated minimal morphological changes in NIH/3T3 (Figure 5a) and Vero cells (Figure 5b) after exposure to the PLEC-coated 2% for 24 h, as indicated by the black arrows (live cells) and red arrows (dead cells). The results were consistent with the low cytotoxicity scores observed in the viability assays. Additionally, the low reactivity grades recorded in the agar diffusion test (Table 1) indicate that the coatings did not induce significant toxicity or adverse reactions, confirming that the membrane coatings were highly biocompatible. The biocompatibility of the PLEC-coated membrane was thoroughly evaluated to confirm their safety for a range of medical applications. Additionally, the ability of the coatings to maintain high cell viability along with the absence of adverse effects in direct-contact tests confirms their potential for use in medical devices requiring long-term contact with tissues. The results of multiple assays verify the suitability of the coatings for applications that require both strong antimicrobial protection and biocompatibility.

**Figure 5:**
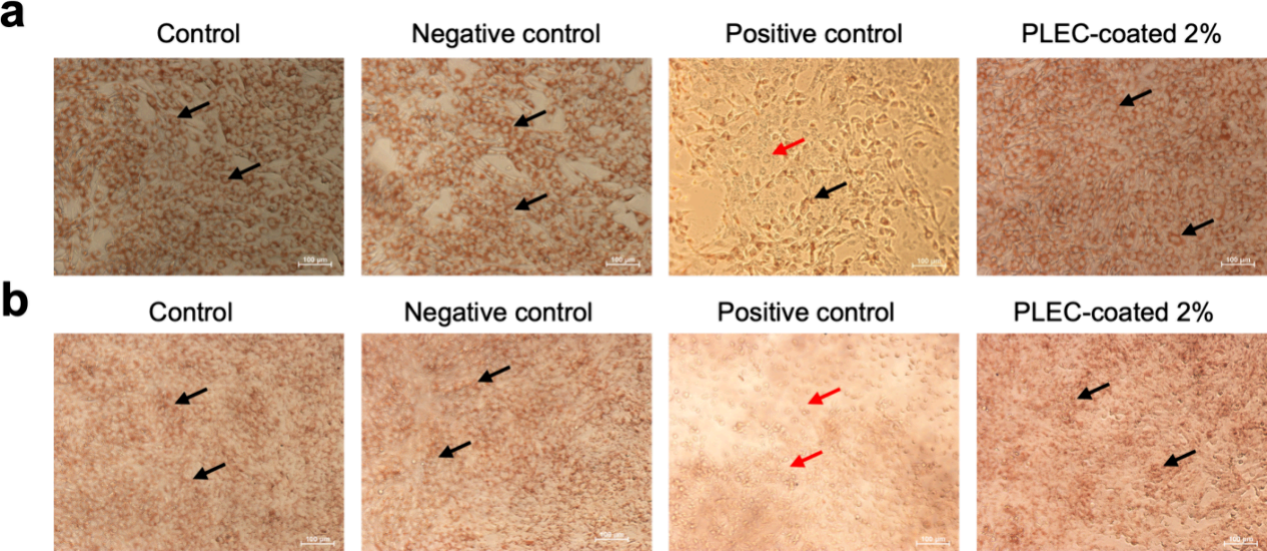
Biocompatibility of the membrane coating. PLEC coating biocompatibility was evaluated through morphological analysis using the agar diffusion test with neutral red staining in (a) NIH/3T3 and (b) Vero cells for 24 h. Microscopic images show morphological changes in both cells after 24 h of exposure to PLEC-coated 2%. Minimal morphological changes evident, suggesting low cytotoxicity. Images taken at 10× magnification, and the reactivity grades for the agar diffusion test are presented in Table 1. Black arrows indicate live cells, and red arrows indicate dead cells. Scale bar: 100 μm.

**Table 1:** Reactivity Grades for the Agar Diffusion and Direct-Contact Tests (ISO 10993-5)

grade	reactivity	description of reactivity zone
0	none	no detectable zone around or under specimen
1	slight	some malformed or degenerated cells under specimen
2	mild	zone limited to area under specimen and <0.45 cm beyond specimen
3	moderate	zone extends 0.45–1.0 cm beyond specimen
4	severe	zone extends >1.0 cm beyond specimen

Beyond confirming cytocompatibility, the combination of natural antimicrobial extracts with polysaccharide-based encapsulation provides additional benefits, including improved environmental compatibility and a lower risk of resistance development. Compared to conventional metal-based antimicrobial systems,^10–13^ the PLEC formulation minimizes concerns related to cytotoxicity and bioaccumulation while preserving broad-spectrum efficacy. These advantages, along with the use of biocompatible components, highlight the system’s suitability for safe integration into biomedical and environmental applications.

### Bacterial Membrane Integrity

TEM was used to examine the bacterial membrane integrity and investigate the mechanism by which PLEC-coated membranes exert antibacterial effects. Figure 6a shows the TEM images of E. coli and S. aureus exposed to PLEC-coated 2% and PLEC-sol 2% for 1 h. The images reveal significant membrane disruption, with PLEC attachment in the bacterial membrane surface of the PLEC-coated 2%, lysis, and bacterial membrane fragmentation in the PLEC-sol 2%-treated group compared with the control and blank groups, which maintained intact membranes. These results indicate that the primary target site of PLEC is the cell membrane, which is the pathway that disrupts structural stability to prevent bacteria growth.

**Figure 6:**
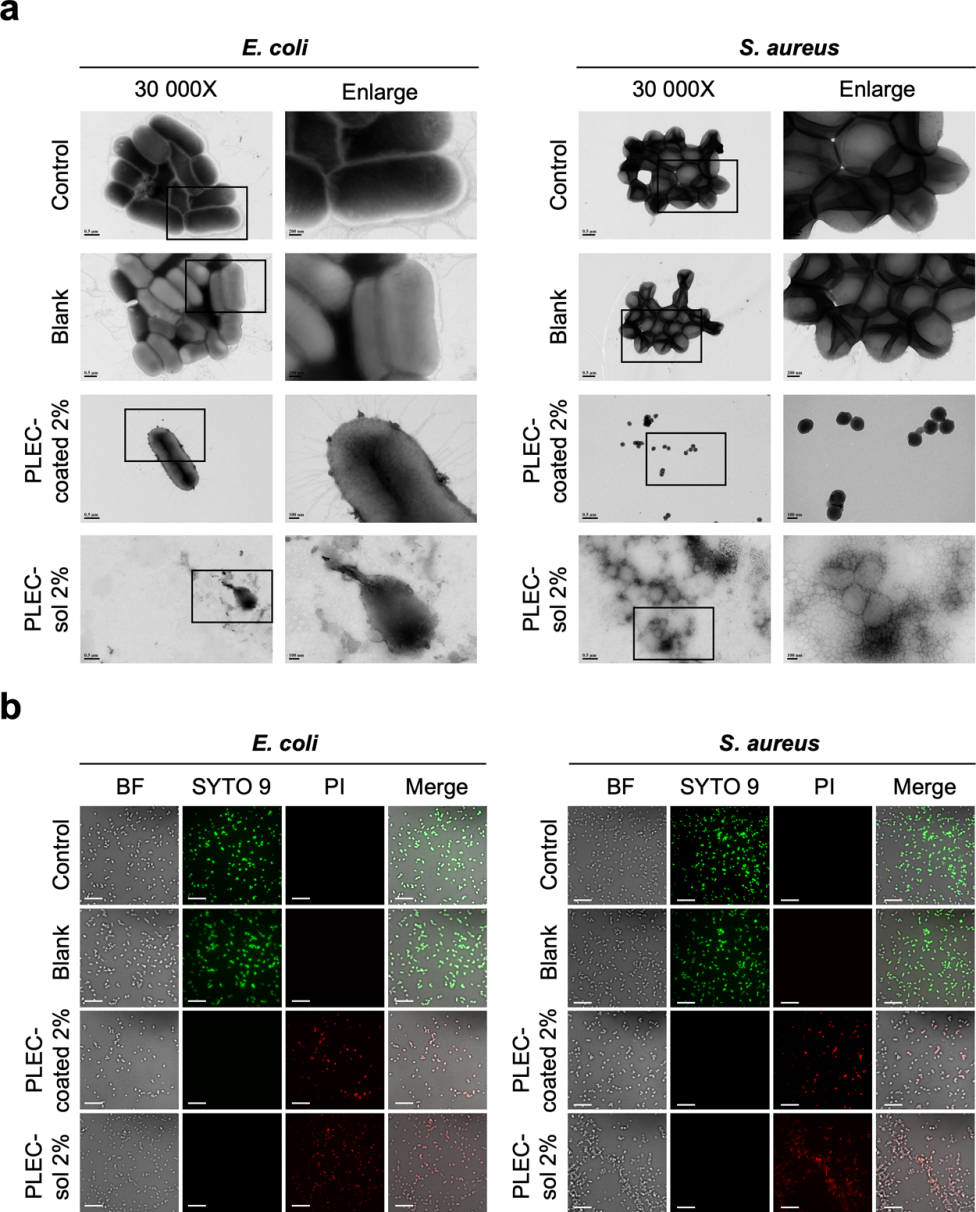
Bacterial membrane integrity assessment. Antibacterial mechanism of the coated membranes was assessed by evaluating their integrity via TEM and live/dead staining. (a) TEM images showing the morphologies of E. coli and S. aureus after 1 h of exposure to PLEC-coated 2% and PLEC-sol 2%. Bacteria exposed to the coatings exhibited considerable membrane damage and lysis compared to those of the control group. (b) Confocal microscopy images of the SYTO 9/PI live/dead staining of E. coli and S. aureus. The control and blank groups predominantly exhibited green fluorescence (live bacteria), whereas the PLEC-coated 2% and PLEC-sol 2% groups exhibit red fluorescence, indicating significant bacterial membrane disruption. Scale bar: 20 μm.

Live/dead bacterial staining with SYTO 9 and PI was performed to confirm the integrity of the bacterial membrane. Confocal microscopy images illustrated the fluorescence signals emitted by live (SYTO 9, green fluorescence) and dead (PI, red fluorescence) cells. In the control and blank groups, most bacteria exhibited green fluorescence, indicating that the cells were alive. Contrastingly, bacteria exposed to PLEC-coated 2% and PLEC-sol 2% exhibited predominantly red fluorescence, indicating membrane damage and cell death. These results suggest that PLEC function by directly interacting with and disrupting bacterial membranes led to the rapid destruction of bacterial cells.^20,21,42,47^

Between 30 min and 1 h after testing, PLEC were released from the coating, after which they attached to and directly destroyed the bacterial cell membranes, as observed via TEM imaging. This process led to ongoing damage to intracellular components owing to the disrupted structural integrity of the bacteria, which was indicated by significant differences in the fluorescence signals among the control, blank, PLEC-coated 2%, and PLEC-sol 2% groups.

## Conclusions

We developed a multifunctional antimicrobial PLEC coating for PP substrates that offers significant improvement with regard to infection control. The PLEC membrane coating exhibited strong antibacterial and antifungal properties, with sustained antimicrobial activity against both Gram-positive and Gram-negative bacteria, as well as fungi. The biocompatibility of the coating ensures its safe application in medical devices, whereas its ability to disrupt bacterial membranes significantly enhances its antimicrobial effectiveness. This innovation provides a promising solution to address the growing challenge of AMR and paves the way for the development of advanced antimicrobial materials for healthcare applications. In the future, we will investigate the long-term stability of the coating and its performance in different models beyond those of PP substrates. This work enhances the durability of coatings and broadens their application, highlighting their potential to significantly advance infection control in healthcare and other fields.

## Supplementary Material



## Notes

Antibacterial performance of PLEC-coated membranes (1%, 2%, 3%) using modified AATCC method; 16 h incubation; initial inoculum 10^7^ CFU/mL; identification of PLEC-coated 2% as minimum inhibitory concentration; and tested strains: E. coli, P. aeruginosa, S. aureus, and S. mitis (PDF)
