# Supplementary material for: Polysaccharide-Encapsulated Lauraceae Extract Complex Coating Conferring Antimicrobial Properties to Polypropylene Surfaces
Source: ACS Omega. 2025 Jun 3;10(23):24422–31. doi: 10.1021/acsomega.5c00665 (PMC12177763; doi:10.1021/acsomega.5c00665)
Supplement: Supplementary file 1 [file ao5c00665_si_001.pdf]

Supplementary Information

Polysaccharide-Encapsulated Lauraceae Extract Complex Coating conferring Antimicrobial Properties to Polypropylene Surfaces

Tuyet-Nhi Do[1], Po-Hsin Lee[2], Tsung-Lin Tsai[1,3,4], Ping Ching Wu[1,3,5,6*]*

[1]Department of Biomedical Engineering, College of Engineering, National Cheng Kung University, Tainan 701, Taiwan

[2]Meet Tec. Co., Ltd., Tainan 710, Taiwan

[3]Center of Applied Nanomedicine, National Cheng Kung University, Tainan 701, Taiwan

[4]Department of Oncology, National Cheng Kung University Hospital, College of Medicine, National Cheng Kung University, Tainan 701, Taiwan

[5]Institute of Oral Medicine and Department of Stomatology, National Cheng Kung University Hospital, College of Medicine, National Cheng Kung University, Tainan 701, Taiwan

[6]Medical Device Innovation Center, Taiwan Innovation Center of Medical Devices and Technology, National Cheng Kung University Hospital, National Cheng Kung University, Tainan 701, Taiwan

Corresponding Authors

* Ping-Ching Wu; E-mail: wbcxyz@gmail.com

* Tsung-Lin Tsai; E-mail: sloantsai@mail.ncku.edu.tw

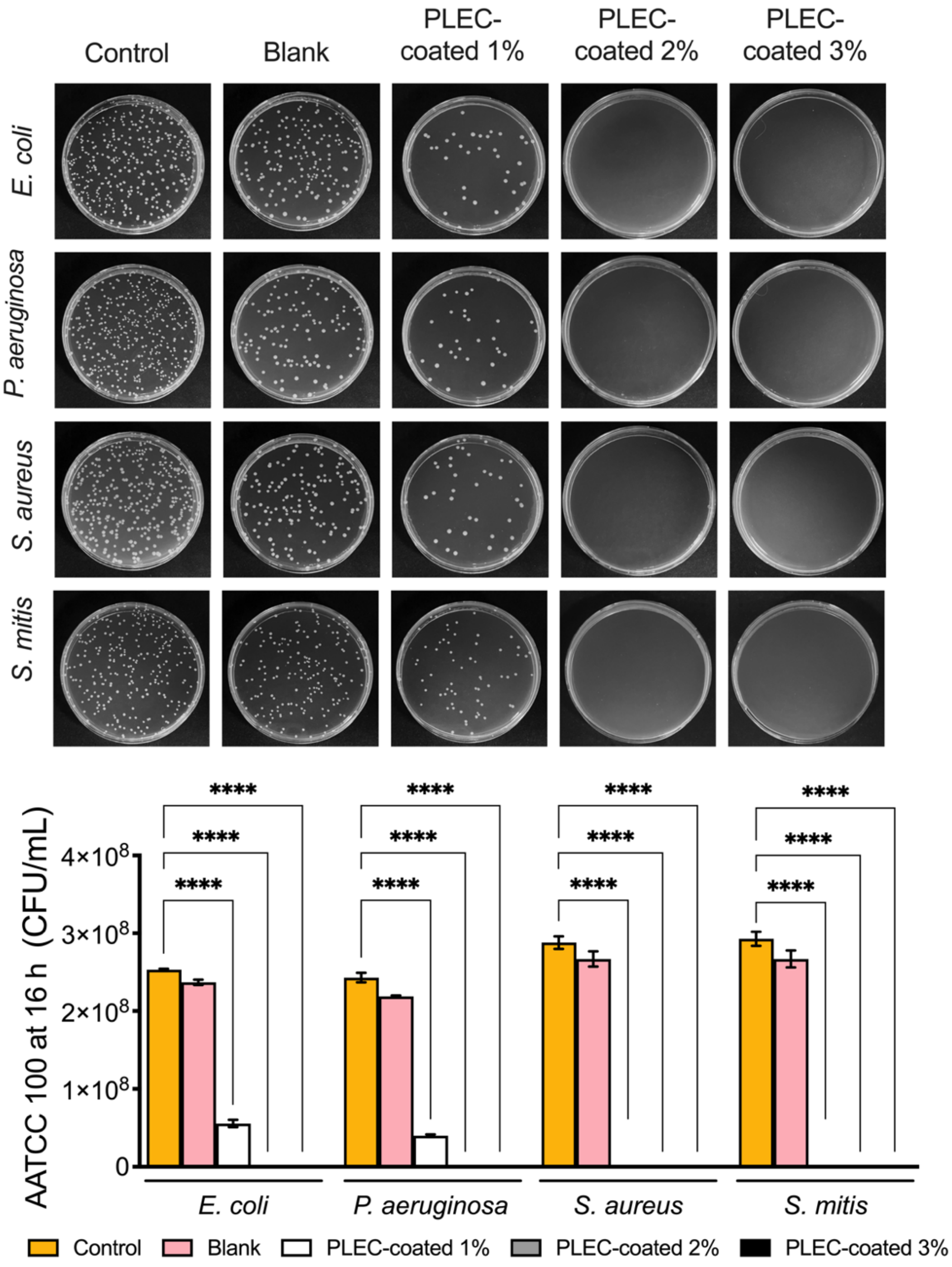

Figure S1. Antibacterial activity of PLEC-coated 1%, 2%, and 3% against *E. coli, P. aeruginosa, S. aureus,* and *S. mitis* (initial inoculum of 10^7 CFU/mL) after incubation for 16 h. PLEC-coated 2% was identified as the minimum inhibitory concentration, effectively limiting bacterial proliferation. Two-way ANOVA results: **** $p < 0.0001$ compared with the control group. Values expressed as mean ± SEM (n = 3).
